# Identification of obesity risk factors in 3-12-year-old children and adolescents with prior respiratory tract infections via interpretable machine and deep learning models

**DOI:** 10.1093/jamiaopen/ooag061

**Published:** 2026-08-06

**Authors:** Xiao-Qian Wang, Fang-Jie-Yi Zheng, Qiong Wang, Che Li, Wen-Qian Zhang, Zhi-Xin Zhang, Wen-Quan Niu

**Affiliations:** Capital Institute of Pediatrics, Chinese Academy of Medical Sciences & Peking Union Medical College, Beijing 100020, China; Center for Evidence-Based Medicine, Capital Center for Children’s Health, Capital Medical University, Capital Institute of Pediatrics, Beijing 100020, China; Graduate School, Beijing University of Chinese Medicine, Beijing 100029, China; Department of Pediatrics, China-Japan Friendship Hospital, Beijing 100029, China; Center for Evidence-Based Medicine, Capital Center for Children’s Health, Capital Medical University, Capital Institute of Pediatrics, Beijing 100020, China; Center for Evidence-Based Medicine, Capital Center for Children’s Health, Capital Medical University, Capital Institute of Pediatrics, Beijing 100020, China; Department of Pediatrics, China-Japan Friendship Hospital, Beijing 100029, China; Institute of Clinical Medical Sciences, China-Japan Friendship Hospital, Beijing 100029, China; Center for Evidence-Based Medicine, Capital Center for Children’s Health, Capital Medical University, Capital Institute of Pediatrics, Beijing 100020, China

**Keywords:** Childhood obesity, respiratory tract infection, machine learning, deep learning, SHapley Additive exPlanations

## Abstract

**Backgrounds and Objectives:**

Childhood obesity and respiratory tract infections (RTIs) are 2 major global public health issues that frequently co-occur and are closely interrelated. Early detection of children with prior RTIs who are at high obesity risk is crucial for targeted interventions. This study integrates interpretable machine learning (ML) models and a deep learning network to develop an obesity risk prediction model in a large pediatric cohort.

**Methods:**

Cross-sectional data from 6509 children and adolescents aged 3-12 years with prior RTIs in Beijing and Tangshan were fed to 12 ML models to predict childhood obesity (versus children with normal weight). Bayesian optimization was applied to fine-tune model hyperparameters. Prediction performance was assessed using 8 metrics. Key predictive features were identified by SHapley Additive exPlanations (SHAP). The validity of the optimal ML model was verified by the sequential neural network model.

**Results:**

Of 12 ML models, LightGBM achieved the optimal performance (accuracy: 0.8844, area under the curve [AUC]: 0.9491). SHAP analysis identified 20 key predictors, including child age, paternal body mass index (BMI), maternal BMI, birth length, birthweight, gestational age, eating speed, complementary feeding initiation age, screen time, breastfeeding duration, bedtime, maternal age, nighttime sleep, outdoor activity, dental caries, sedentary time, food allergies, family history of diabetes, and sex. The deep learning sequence network model further validated the predictive value of these features (accuracy: 0.8023, AUC: 0.8117), and the SHAP-driven feature importance rankings were in close line with LightGBM.

**Conclusions:**

Our LightGBM-based model enables effective prediction of obesity risk in children aged 3-12 years with prior RTIs, and the key features identified can inform early screening and facilitate personalized interventions.

## Introduction

Childhood obesity has become one of the most pressing global public health challenges, with its prevalence rising rapidly in both developed and developing countries.^1^ The World Health Organization (WHO) reports that the number of children and adolescents aged 5-19 years living with obesity globally surged from 11 million in 1975 to 340 million in 2023.^2^ In China, the 2020 National Nutrition and Chronic Disease Survey showed that the combined prevalence of overweight and obesity was 10.4% in children under 6 and 19% in those aged 6-17 years. It is estimated that Chinese children aged 0-19 are expected to bear substantial losses in disability-adjusted life years, with an estimated economic burden of $31.6 trillion and an average societal cost of $350 000 per affected child.^3^ Clinically, childhood obesity is associated with insulin resistance, hypertension, and psychosocial impairments.^4–6^ It is increasingly regarded as a pathological condition rather than merely a consequence of unhealthy lifestyles, and represents a key modifiable risk factor for numerous comorbidities, especially respiratory tract infections (RTIs).

RTIs are among the most prevalent pediatric disorders, characterized by high incidence, diverse etiologies, recurrent episodes, and prolonged recovery periods,^7^^,^^8^ and they severely compromise children’s physical and mental health^9^^,^^10^ and constitute a major health background for children at enhanced obesity risk. Notably, the latest Global Burden of Disease (GBD) study identified lower RTIs as the leading cause of death in children under 5. An 80-year follow-up cohort study revealed that over 20% of premature respiratory-related deaths in adults were directly attributable to lower RTIs during early childhood. Epidemiological evidence suggests that children and adolescents with a history of RTIs may be more susceptible to obesity.^11^ Potential underlying mechanisms include RTI-induced disruptions in metabolic homeostasis, alterations in dietary and physical activity patterns during recovery, or induction of chronic low-grade inflammation, all of which may contribution to weight gain. Conversely, obesity can impair respiratory function and increase RTI susceptibility, forming a bidirectional relationship that exacerbates health risks.^12^^,^^13^ However, the specific factors influencing obesity development in children and adolescents with prior RTIs remain poorly understood, representing a big gap in pediatric research.

It is widely recognized that obesity impacts respiratory health in children. In a study of 143 hospitalized children aged 1-5 years with acute RTIs, those with obesity had longer hospital stays, greater requirements of oxygen therapy, and higher rates of mechanical ventilation than their normal-weight counterparts.^14^ Other studies have demonstrated an inverse association between pulmonary oxygen uptake capacity and body mass index (BMI), with children with obesity exhibiting poorer baseline respiratory function and enhanced RTI susceptibility.

Currently, most prior studies have adopted conventional statistical methods to investigate factors associated with childhood obesity.^15^^,^^16^ Although these approaches can model complex nonlinear interactions among multiple risk factors, advanced analytical techniques offer substantial advantages. For example, machine learning (ML) enables the detection of complex patterns in high-dimensional data and identification of subtle risk characteristics; however, its limited transparency raises concerns in clinical practice and hinders translational application.

To produce more information, the present study integrated multiple interpretable ML models with a deep-learning neutral network to develop and validate an obesity risk prediction model among children and adolescents with prior RTIs from Beijing and Tangshan.

## Methods

### Study design and participants

This survey used a cross-sectional cluster sampling design and was conducted in Beijing and Tangshan, Hebei Province, between September 2020 and January 2022. The survey protocol adhered to the Declaration of Helsinki.

The first data collection wave was carried out from September to December 2020. Preschool-aged children were sampled from 4 of 16 districts in Beijing and 2 of 7 districts in Tangshan, with 5 kindergartens selected in each district (30 kindergartens in total). The second wave was in January 2022 among 8 primary schools in Pinggu District, Beijing.

Self-designed questionnaires were initially distributed to parents or guardians of 18 503 children and adolescents. After excluding 3519 children outside the 3-12-year age range, 14 984 children remained. An additional 1707 underweight and 2410 overweight children were then excluded, leaving 10 867 participants. Finally, 4358 children and adolescents without a reported history of RTIs were excluded, leaving 6509 children and adolescents with prior RTIs included in the final analysis.

### Data collection

Data were collected using self-designed questionnaires. Prior to formal distribution, the reliability and validity of the questionnaire were assessed through a pre-test on 200 participants. The internal consistency was satisfactory, with Cronbach’s α coefficients exceeding 0.85.

Analytical data covers 5 domains: (1) Child demographic information: age (years), sex, and nationality; (2) Maternal pregnancy, childbirth, and early life factors: birthweight (grams), birth length (centimeters), gestational age (weeks), delivery mode, reason for caesarean section, birth order (induced abortion, spontaneous abortion, and normal pregnancy), maternal pregnancy order, twin status, feeding method within the first 6 months of life, breastfeeding duration (months), and age at introduction of complementary foods (months); (3) Child health status: food allergies, drug allergies, and number of decayed teeth; (4) Lifestyle-related factors: frequency of fast food consumption, frequency of sweet food consumption, frequency of late-night snacking, outdoor time (hours), sedentary time (hours), nighttime sleep duration (hours), eating speed (minutes), and screen time (minutes); and (5) Household characteristics: maternal age (years), paternal age (years), paternal educational attainment, maternal educational attainment, maternal BMI, paternal BMI, number of family members with diabetes, and annual household income.

### RTI definition

The questionnaire also gathered information on children’s history of both upper and lower RTIs. Upper RTIs include colds, rhinitis, pharyngitis, and tonsillitis; lower RTIs include tracheitis/bronchitis and pneumonia. The number of episodes of each RTI in the past year was recorded. Children and adolescents with no episodes of either upper or lower RTIs were classified as having no history of RTIs.

### Obesity definition

BMI was calculated as weight in kilograms divided by height in meters squared (kg/m^2^). The classification of different weight status categories was determined using children’s age-and sex-standardized BMI z-scores, calculated as the deviation of a child’s BMI from the population mean in standard deviations (SDs). Based on the 2006 WHO growth standards for children aged 0-5 years^17^ and the 2007 WHO growth reference for school-aged children aged 5-19 years (61-228 months), obesity as a BMI z-score > +2 SD.

### Quality control

Given that the survey was administered online, ensuring data quality was essential. Prior to questionnaire distribution, trained personnel provided detailed explanations of all items to designated teachers, who subsequently disseminated the instrument to parents or guardians. During questionnaire completion, teachers consulted trained staff for clarification when necessary. The submitted data were exported from the “Wenjuanxing” free platform (https://www.wjx.cn/) into Microsoft Excel™ files and subjected to systematic review by trained personnel. Cases involving missing information or outliers were returned to the responsible teachers, who requested verification or supplementation from parents or guardians.

### Statistical analysis

After checking the normality of continuous variables, those with normal distributions are expressed as mean ± SD, and with skewed distributions as median (interquartile range). Categorical variables are expressed as count and percent. Normally-distributed continuous variables were compared using Student’s t-test, and skewed continuous variables using the Mann–Whitney U test. Categorical variables were compared using the Fisher’s exact test or χ^2^ test, where appropriate. A 2-tailed *P*-value < .05 indicates statistical significance.

The data preprocessing workflow consisted of 4 sequential steps: (1) Missing value imputation: continuous variables with missing values were imputed using the mean, and categorical variables using the mode; (2) Collinearity reduction: For highly correlated feature pairs (Spearman’s correlation coefficient |ρ| ≥ 0.7), 1 variable was excluded based on lower clinical relevance; (3) Class imbalance handling: The Synthetic Minority Over-sampling Technique (SMOTE) was used to generate synthetic samples, thereby balancing the class distribution between normal-weight and obese children and adolescents; and (4) Data splitting: The dataset was randomly divided into a training set, a test set, and a validation set at a ratio of 7:2:1.

Twelve ML models were trained on the training set. Model performance was assessed, and hyperparameters were tuned on the validation set. The optimal model was applied to the independent test set to evaluate its generalization ability and robustness on new data. The models included Adaptive Boosting (AdaBoost), Decision Tree, Extra Random Trees, Extreme Gradient Boosting (XGBoost), Gradient Boosting, K-Nearest Neighbor (KNN), Light Gradient Boosting Machine (LightGBM), Linear Discriminant Analysis (LDA), Logistic Regression, Naive Bayes, Quadratic Discriminant Analysis (QDA), and Random Forest. Hyperparameters were tuned via Bayesian optimization combined with 5-fold cross-validation to ensure model stability. The optimal model was selected based on 8 evaluation metrics: 2 primary metrics (accuracy and area under the receiver operating characteristic [ROC] curve [AUC]) and 6 secondary metrics (sensitivity, specificity, positive predictive value [PPV], negative predictive value [NPV], F1-score, and Youden’s index).

The selected variables were fed into a deep sequential neural network to further evaluate their predictive performance. This network had 2 hidden layers, with the dataset partitioned into training and validation sets at a 7:3 ratio. Grid search was used for hyperparameter tuning to optimize model performance, and 5-fold cross-validation was used to assess model generalization ability. The same 8 aforementioned metrics were used to evaluate the performance of the deep sequential neural network.

To enhance the interpretability of both ML models and deep sequential neural network, SHapley Additive exPlanati (SHAP) was used. Based on Shapley values derived from game theory, this method quantifies each feature’s contribution to the model’s prediction outcome, converting opaque decision-making processes into transparent, interpretable feature importance scores. By calculating SHAP values, a global model interpretation was obtained to identify key features driving overall obesity risk prediction, and a comparative assessment of feature importance to predict obesity by comparing SHAP rankings across the optimal ML model and the deep sequential neural network. Local SHAP explanations offer deeper insights into the model’s reasoning for individual sample predictions.

The analytical pipeline was implemented using PyCharm (Community Edition 2024.3.4 x64) embedded with Python (Python Software Foundation, Version 3.6.1) under the Windows 10 operating system. Data balancing via SMOTE was performed using the DMwR package in the R coding platform (version 4.3.3).

### Ethical considerations

Given the 2-wave design of this study, ethical approval was obtained separately from the Ethics Committee of the China–Japan Friendship Hospital and Beijing University of Chinese Medicine. Parents or guardians provided written informed consent. All data collection procedures adhered to local data protection requirements, with all study data stored on an access-restricted server accessible exclusively to the research team. To ensure participant confidentiality, personally identifiable information was anonymized by assigning unique identifiers before data analysis. This study was reported in line with the STROBE (Strengthening the Reporting of Observational Studies in Epidemiology) guidelines.

## Results

### Baseline characteristics

Table S1 shows the baseline characteristics of 6509 children and adolescents with prior RTIs. Of these, 5291 were classified as having normal weight, and 1218 as obese.

Figure S1 depicts the comparative distribution of normal-weight and obese children and adolescents before and after SMOTE-based inter-group balancing. Figure S2 displays the distribution of each characteristic. Analysis of the association between individual characteristics and childhood obesity revealed minimal between-group differences in obese and normal-weight children and adolescent across all evaluated characteristics (Figure S3).

### Optimal ML model selection

Comparisons of continuous (Figure S4) and categorical (Figure S5) variables before and after missing imputation revealed no substantial distributional changes, indicating the coherence of the imputed datasets. After separate imputation, Spearman’s correlation analysis was performed to remove weakly associated features. Two highly correlated pairs were identified: child age vs fast-food frequency (ρ = -0.709) and parental age vs maternal age (ρ  =  0.839). Fast-food consumption frequency was retained due to its stronger association with childhood obesity (r = 0.241 vs r = 0.213 for age); maternal age was preserved given its relatively higher clinical relevance (correlation with obesity: 0.066 vs 0.058). All other feature pairs exhibited correlation coefficients below 0.7 (Figure S6). The SMOTE-processed dataset was then split into training, validation, and test sets at a 7:2:1 ratio.

**Figure 1:**
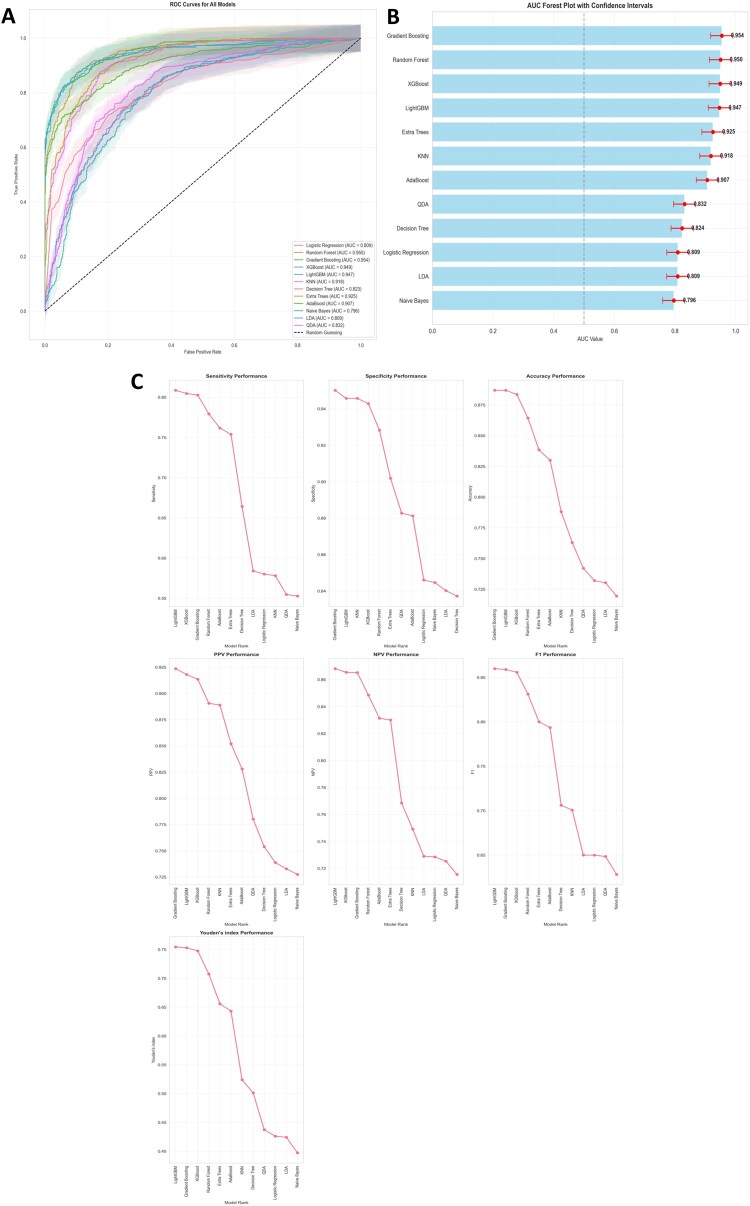
Performance Comparison of 12 Machine Learning Models using ROC Curves (Panel A), AUC Forest Plot with 95% Confidence Interval (Panel B), and Comparison of Other Performance Metrics (Panel C).

Twelve ML models were evaluated using 8 metrics. For binary classification tasks, AUC is recognized as the most robust and widely used evaluation metric. Based on the AUC values, Gradient Boosting ranked first (0.9557), followed by Random Forest (0.9503), LightGBM (0.9491) and XGBoost (0.9449). ROC curves and AUC rankings with 95% confidence intervals are presented in Figure 1A and B.

Gradient Boosting and LightGBM outperformed Random Forest across most secondary metrics; for example, accuracy was 0.8869 for Gradient Boosting, 0.8844 for LightGBM, and 0.8643 for Random Forest. Compared to XGBoost, Gradient Boosting and LightGBM achieved higher AUC values, while performance on the remaining 7 metrics was marginally low; for example, F1-score was 0.8598 for Gradient Boosting, 0.8571 for LightGBM, and 0.8640 for XGBoost. The remaining 9 models exhibited inferior performance than Gradient Boosting and LightGBM. Comprehensive comparisons of all evaluation metrics are shown in Figure 1C, with detailed numeric results listed in Table S2.

Calibration curves (Figure S7A) showed that LDA and logistic regression were closest to the ideal reference line, followed by XGBoost, decision tree, extra trees, and LightGBM. Clinical impact curves (Figure S7B) indicated that both gradient boosting and LightGBM had larger positive AUC values, confirming strong robustness and clinical utility. Confusion matrices for all models are present in Figure S8.

LightGBM was selected as the optimal ML model for 3 key reasons: (1) it represents an optimized implementation of Gradient Boosting, inheriting its core algorithmic principles; (2) it delivers comparable predictive performance to Gradient Boosting; and (3) it offers substantial advantages in training speed, computational efficiency, and memory usage.

### Optimal ML model interpretation

The optimal LightGBM model was interpreted via SHAP from both global and local perspectives. Global feature importance was ranked in descending order of average SHAP values (Figure 3A), identifying the top 20 features influencing obesity risk prediction in children and adolescents with prior RTIs: child’s age, paternal BMI, maternal BMI, birth length, birthweight, gestational age, eating speed, complementary feeding initiation age, screen time, breastfeeding duration, bedtime, maternal age, nighttime sleep, outdoor activity, dental caries, sedentary time, food allergies, family history of diabetes, and sex.

The SHAP swarm plot (Figure 3B) revealed that among modifiable lifestyle factors, children and adolescents with prior RTIs were more susceptible to obesity if they had faster eating speed, longer screen time, shorter outdoor activity duration, shorter nighttime sleep duration, fewer dental caries, and longer sedentary time.

To clarify the model’s individualized prediction process, local SHAP analyses were conducted using waterfall plots for individual cases in the validation set (Figure 4A and B). Pink bars denote features increasing the predicted obesity probability, while blue bars indicate features decreasing this probability; bar length reflects the magnitude of each feature’s contribution to the model’s final output. F(x) represents the model’s predicted probability for a specific individual, and E[f(x)] represents the model’s average output value in the absence of feature inputs. For an obese child (Figure 4A), the predicted probability was 0.8823. Eating speed, screen time, and birth length mitigated the increase in predicted probability, while maternal BMI, drug allergies, paternal BMI, sex, and age promoted an elevated prediction probability. Specifically, for modifiable features (denoted by pink bars), clinical interventions could be tailored; for example, guiding the child and their parents to engage in moderate physical activity to reduce weight and alter obesity status. For a non-obese child (Figure 4B), the predicted obesity probability was 0.1030. Bedtime, age, drug allergies, sedentary time, and food allergies reduced the predicted probability, while paternal BMI, sleep duration, birth length, and paternal educational attainment increased it. Integrating global SHAP insights with modifiable features, moderate increases in the child’s nighttime sleep duration are recommended to maintain a healthy BMI.

The SHAP decision graph (Figure 4C) visualizes individual prediction trajectories. Each line depicts an individual’s predictive process. Feature importance is ranked from top to bottom, with features ranked by importance from top to bottom (topmost features exert the greatest influence on outcomes). Lines extending to the right indicate features increasing predicted obesity probability, while leftward extensions denote decreasing probabilities. For the representative individual shown, the final predicted obesity probability ranged between 0.525 and 0.550; age, birth weight, birth length, eating speed, bedtime, outdoor activity duration, and gestational age increased the prediction probability, while screen time, age at introduction of complementary foods, nighttime sleep duration, maternal BMI, number of family members with diabetes, and breastfeeding duration reduced it.

The SHAP dependency heatmap for the optimal LightGBM model (Figure S9) clarifies feature contributions to individual predictions across the cohort. Each column represents an individual; observing the color across an entire column indicates whether a feature promotes (red) or diminishes (blue) the predicted disease status for that sample. Vertically, the heatmap quantifies each feature’s importance in the prediction process for an individual, while horizontally, it reveals variability in a feature’s influence across different individuals.

### Performance assessment using deep sequential neural network

**Figure 2:**
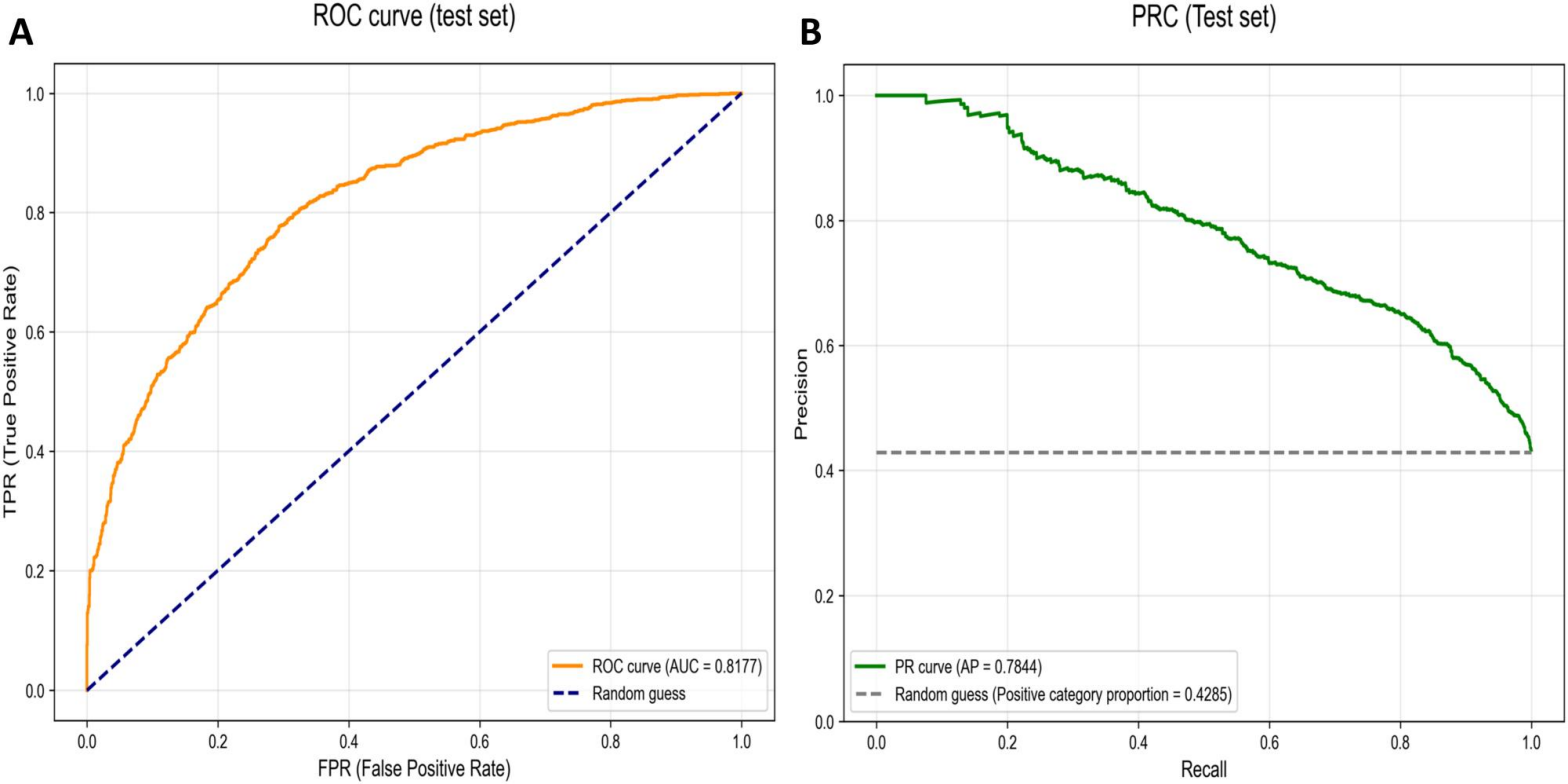
ROC curve (Panel A) and PRC curve (Panel B) of the Sequential Neural Network Model on the Test Set.

The predictive performance and SHAP-derived insights of the deep sequential neural network (trained on the 20 key features identified by ML models) were compared with those of the optimal LightGBM model. Evaluation using the 8 core metrics (Table S3) demonstrated that the deep sequential neural network achieved satisfactory performance in accuracy (0.8023) and AUC (0.8177), albeit slightly lower than the LightGBM model (accuracy: 0.9413; AUC: 0.9491). The AUC and Precision-Recall Curve (PRC) for the deep sequential neural network are presented in Figure 2.

**Figure 3:**
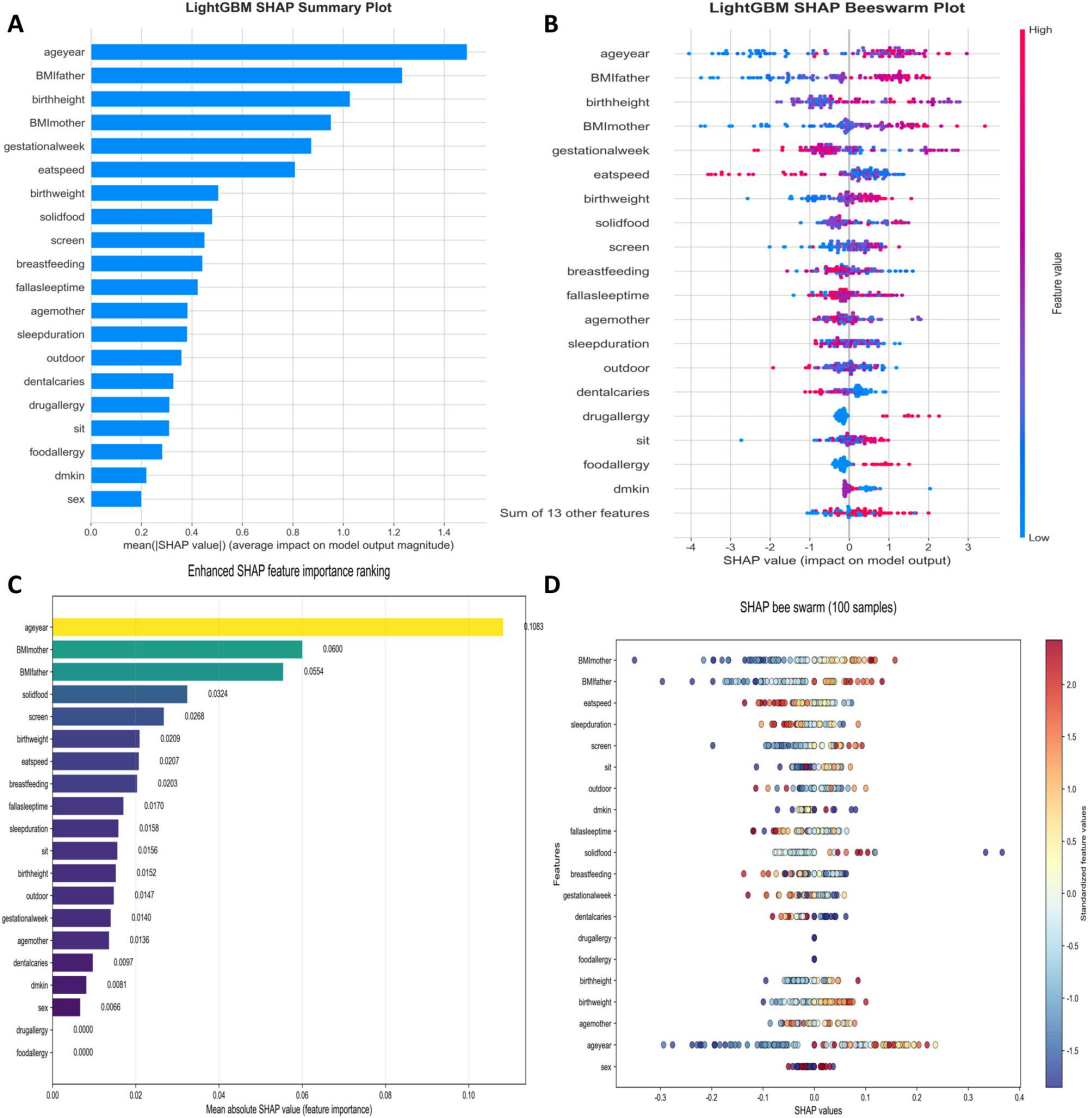
Global SHAP Interpretation of Features Predicting Childhood Obesity under the Optimal LightGBM Model and Sequential Neural Network Model using SHAP Mean Ranking (Panel A and Panel C) and Bee Swarm Plot (Panel B and Panel D).

The ranking of average SHAP values for the deep sequential neural network (Figure 3C) was broadly consistent with that of the LightGBM model (Figure 3A), with the exception that food allergies and drug allergies exhibited a SHAP value of 0 (ie, no predictive contribution) in the deep learning model. The direction of each feature’s influence on obesity prediction, as visualized in the SHAP swarm plot of the deep sequential neural network (Figure 3D), was also consistent with the LightGBM model (Figure 3B). These findings collectively confirm the strong and consistent predictive power of the 20 features identified by ML models.

**Figure 4:**
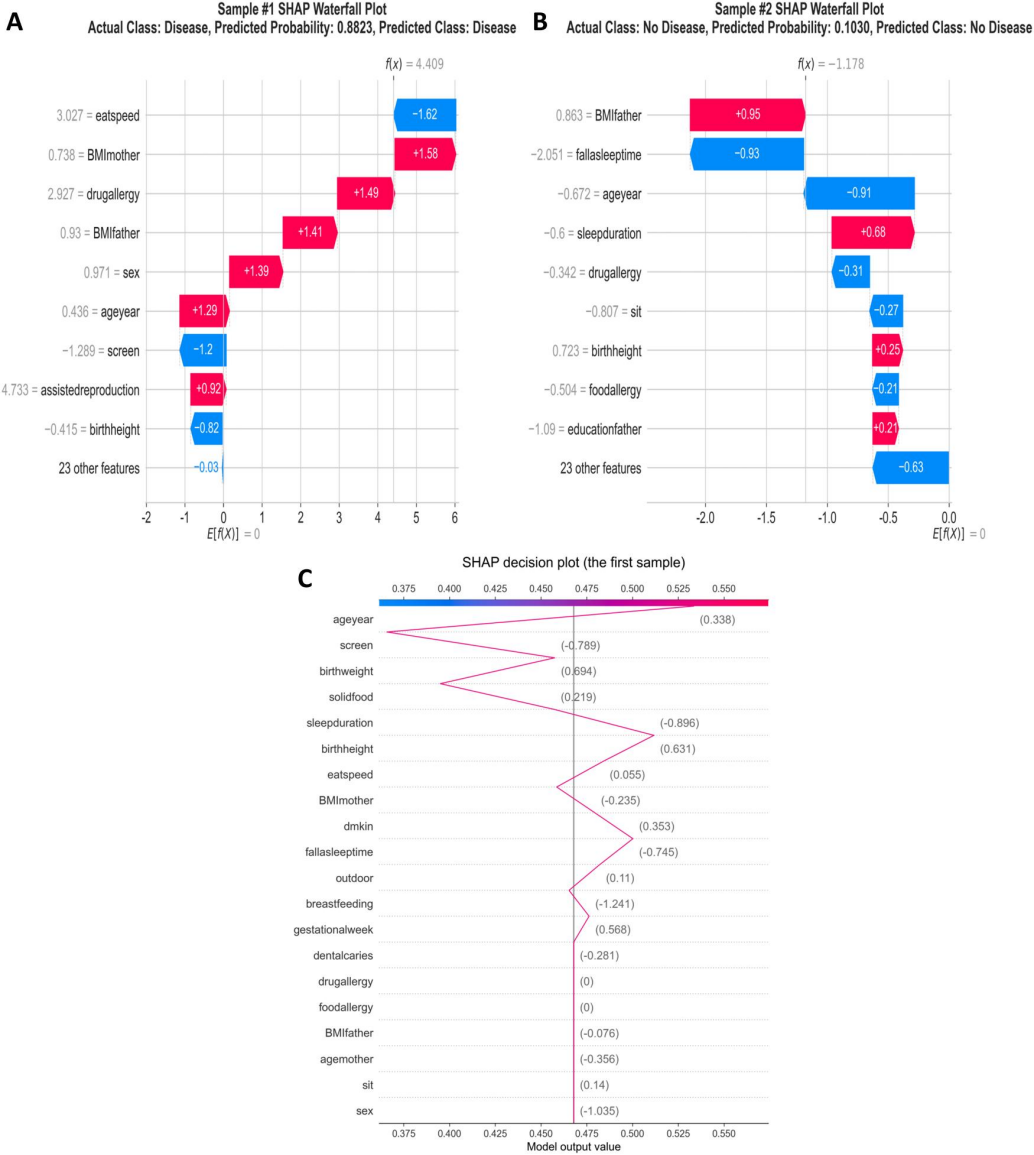
SHAP Local Interpretability Waterfall Plots (Panels A and B) and Decision Plot (Panel C) Displaying Childhood Obesity under the Optimal LightGBM Model Using Machine Learning and Sequential Neural Network Models.

## Discussion

This study developed and compared multiple ML models to predict obesity risk among children and adolescents aged 3-12 years with prior RTIs, with the aim of identifying the optimal model. Notably, LightGBM outperformed other models, and its predication performance was verified using multiple evaluation metrics and further validated by a deep sequential neural network. Moreover, SHAP analysis was applied to screen and visualize a total of 20 key features. To our knowledge, this is the first study to combine ML for feature screening and deep learning for external validation, thereby enhancing both model robustness and the reliability of feature selection. This framework provides a novel strategy for obesity risk prediction and the development of personalized interventions in children and adolescents with prior RTIs.

Early management and targeted intervention for childhood obesity represent a priority in children and adolescents with prior RTIs, given the well-documented bidirectional interplay between RTIs and pediatric obesity.^18^^,^^19^ A spectrum of respiratory manifestations, including coughing, dyspnea, and chest distress, often persist for weeks to months following acute infection; in some cases, these symptoms fail to solve despite apparent clearance of the initial infection insult, ultimately progressing to chronic respiratory morbiodity.^20^^,^^21^ Chronic respiratory symptoms impose substantial functional limitations, constraining physical activity participation, prolonging sedentary behavior, and impairing both sleep quality and total sleep duration.^22^ Cumulatively, these behavioral and physiological disruptions accelerate the onset of excessive weight gain over time. Existing research demonstrates that obesity increases the risk of adverse respiratory symptoms in school-age children, even in the absence of documented prior RTIs.^23^^,^^24^ Excessive adiposity exerts deleterious effects on both static and dynamic respiratory function, with measurable impairments across key physiological indices including lung capacity, lung compartment mechanics, airway patency and function, and exercise tolerance.^25^^,^^26^ Additionally, obesity is associated with attenuated therapeutic responses to sleep apnea interventions, suggesting that delayed weight management may exacerbate respiratory control deficits and heighten susceptibility to obesity-related hypoventilation and subsequent secondary complications.^27^^,^^28^ Consequently, the present study aims to develop and validate optimal ML models to identify a minimal predictive features set capable of stratifying obesity in this high-risk pediatric cohort. This framework is designed to facilitate early, targeted screening and inform personalized intervention strategies, thereby enabling timely, precision-focused clinical management of pediatric obesity in children with prior RTIs.

Following comparative assessment of multiple evaluation metrics, LightGBM was selected as the optimal model for predicting obesity in children and adolescents with prior RTIs. Theoretically, LightGBM is a high-efficiency gradient boosting algorithm suitable for large-scale datasets and high-dimensional features; a deep sequential neural network was additionally applied to verify the predictive potency of the screened feature set. Deep sequential neural networks contain hidden layers to progressively extract higher-level data representations, enabling capture of latent underlying patterns.^29^^,^^30^ Valuation of the deep learning network and SHAP comparisons revealed minimal discrepancies in feature important rankings, reinforcing that the filtered features possess robust predictive efficacy for childhood obesity.

Via LightGBM-based SHAP interpretative analyses, multiple modifiable lifestyle behaviors were verified to exert strong predictive effects, including eating speed, screen time, outdoor activity time, nighttime sleep duration, and sedentary time were confirmed to have strong predictive power, consistent with prior findings. Notably, children and adolescents with prior RTIs may exhibit heightened susceptibility to unhealthy behavioral patterns, driven by reduced physical activity, sleep disturbances, or disrupted daily routines, which may further exacerbate the impacts of these factors on obesity risk. A considerable proportion of affected children also present with sleep-disordered breathing or fragmented sleep, which are closely linked to daytime fatigue, appetite dysregulation, and heightened obesity susceptibility.^31^ Reduced outdoor activity and poor sleep quality interact synergistically with prolonged screen time, accelerated eating speed, and consumption of high-energy foods, forming behavioral profiles that promote excessive weight gain.^32^ Sustained exposure to such behavioral patterns fosters entrenched detrimental lifestyle habits in children, driving a pronounced increase in obesity risk; these adverse health outcomes often persist into adulthood, predisposing individuals to severe chronic comorbidities including cardiovascular disease, type 2 diabetes mellitus, and hypertension.^33^^,^^34^ Moreover, our findings highlight that early-life factors serve as significant predictors of positive RTI history in pediatric populations. Greater birth length and higher birthweight are associated with a higher probability of childhood obesity, consistent with a meta-analysis revealing that low birth weight correlates with reduced long-term overweight risk, while high birth weight confers a higher likelihood of later-life overweight.^35^ Birth weight and prolonged gestational age typically exert direct impacts on postnatal body composition development and BMI elevation throughout childhood and adolescence, indicating that early growth velocity and intrauterine conditions are direct contributing factors to pediatric obesity.^36^

This study carries several limitations that warrant consideration. First, all enrolled participants were recruited from Beijing and Tangshan using a cross-sectional design, so caution must be exercised when generalizing the findings to other geographic regions or demographic populations. Second, constrained by questionnaire design, weight status was solely evaluated using BMI, without incorporation of supplementary anthropometric indices such as waist circumference or hip circumference. Third, the predictive models incorporated only 33 input features, meaning other unmeasured relevant variables may not have been captured in the analysis. Fourth, inherent uncertainties exist within the questionnaire data: reliance on self-reported RTI history may reduce the accuracy of identifying prior respiratory infections, and recall bias stemming from imprecise participant recollection could introduce measurement errors in completed survey responses. Fifth, external validation of the model has not yet been performed, which limits the robustness and generalizability of the study conclusions.

## Conclusions

This study established a predictive model for obesity in children and adolescents aged 3-12 years with prior RTIs based on a cohort of 6509 pediatric subjects. The LightGBM model demonstrated superior performance relative to other ML models, with enhanced accuracy and AUC values. SHAP analysis was utilized to identify core predictive features associated with obesity risk, which were subsequently applied to train a deep sequential neural network. Follow-up SHAP assessment further validated that the screened 20 key features exhibited stable importance rankings and sufficient predictive capacity for identifying childhood obesity in this high-risk population.

## Supplementary Material

ooag061_Supplementary_Data

## Data Availability

The data underlying this article are available in the Dryad, at https://datadryad.org/10.5061/dryad.gf1vhhn44.
