# Supplementary material for: Identification of obesity risk factors in 3-12-year-old children and adolescents with prior respiratory tract infections via interpretable machine and deep learning models
Source: JAMIA Open. 2026 Aug 6;9(4):ooag061. doi: 10.1093/jamiaopen/ooag061 (PMC13445979; doi:10.1093/jamiaopen/ooag061)
Supplement: Supplementary file 1 [file ooag061_supplementary_data.docx]

**Supplementary Materials**

**Table S1.** Baseline Characteristics of Study Children by Obesity Status.

| **Characteristic** | **Normal-weight controls** | **Children with obesity** | ***P-value*** |
| --- | --- | --- | --- |
|  | **(N=5291)** | **(N=1218)** |  |
| **Demographic information** |  |  |  |
| Age (years) | 5.41 (4.25−7.58) | 7.75 (5.58−9.50) | <0.001 |
| Girls | 2671 (50.48) | 714 (58.62) | <0.001 |
| Nationality |  |  | 0.012 |
| Han | 4841 (91.49) | 1141 (93.68) |  |
| Non-Han | 450 (8.51) | 77 (6.32) |  |
| **Foetal and early life factors** |  |  |  |
| Production mode |  |  | <0.001 |
| Natural childbirth | 2831 (53.51) | 548 (44.99) |  |
| Caesarean section | 2451 (46.32) | 669 (54.93) |  |
| Forceps delivery | 9 (0.17) | 1 (0.08) |  |
| Reasons for caesarean section |  |  | 0.667 |
| Self-imposed | 5184 (97.98) | 1191 (97.78) |  |
| Due to medical necessity | 107 (2.02) | 27 (2.22) |  |
| Number of children |  |  | 0.612 |
| 1-2 | 1450 (89.78) | 265 (90.75) |  |
| ≥3 | 165 (10.22) | 27 (9.25) |  |
| Child’s birth order in the family |  |  | 0.666 |
| 1-2 | 1701 (66.84) | 365 (65.88) |  |
| ≥3 | 844 (33.16) | 189 (34.12) |  |
| Twins |  |  | 0.163 |
| Yes | 5175 (97.81) | 1199 (98.44) |  |
| No | 116 (2.19) | 19 (1.56) |  |
| Infant feeding practices |  |  | <0.001 |
| Breastfeeding | 2875 (54.34) | 624 (51.23) |  |
| Mixed feeding with breast milk and formula | 2034 (38.44) | 459 (37.68) |  |
| Formula feeding | 382 (7.22) | 135 (11.08) |  |
| Birth weight (cm) | 3300 (3000−3600) | 3500 (3100−3750) | <0.001 |
| Birth height (g) | 50 (50−52) | 51 (50−52) | <0.001 |
| Gestational age (weeks) | 39 (38−40) | 39 (38−40) | 0.0281 |
| Breastfeeding duration (months) | 12 (6−18) | 10 (0−15) | <0.001 |
| Add complementary feeding time (months) | 6 (6−6) | 6 (6−7) | 0.1986 |
| **Health status** |  |  |  |
| Food allergy |  |  | 0.003 |
| Yes | 665 (12.57) | 192 (15.76) |  |
| No | 4626 (87.43) | 1026 (84.24) |  |
| Drug allergy |  |  | 0.032 |
| Yes | 282 (5.33) | 84 (6.90) |  |
| No | 5009 (94.67) | 1134 (93.10) |  |
| Dental caries |  |  | <0.001 |
| 0 | 2345 (44.32) | 588 (48.28) |  |
| 1 | 677 (12.80) | 174 (14.29) |  |
| 2 | 848 (16.03) | 210 (17.24) |  |
| 3 | 467 (8.83) | 95 (7.80) |  |
| 4 | 416 (7.86) | 69 (5.67) |  |
| ≥5 | 538 (10.17) | 82 (6.73) |  |
| **Lifestyle-related factors** |  |  |  |
| Outdoor activity time (hours/day) | 1.29 (1−2) | 1.29 (1−2) | <0.001 |
| Sitting duration (hours/day) | 2.86 (1.5−5) | 4.29 (2−6.29) | <0.001 |
| Screen time (hours/day) | 1 (0.64−1.57) | 1.29 (0.79−1.86) | <0.001 |
| Sleep duration (hours/day) | 9.5 (9−10.29) | 9.29 (8.57−10) | <0.001 |
| Eating speed (minutes) | 18.33 (15−25) | 16.67 (13.33−20) | <0.001 |
| Frequency of consuming fast food and fried foods |  |  | <0.001 |
| Every day | 685 (12.95) | 295 (24.22) |  |
| 3 times or more per week | 975 (18.43) | 420 (34.48) |  |
| 1−2 times per week | 1248 (23.59) | 220 (18.06) |  |
| Hardly ever eat | 2383 (45.04) | 283 (23.23) |  |
| Frequency of consuming sweet foods |  |  | <0.001 |
| Every day | 515 (9.73) | 157 (12.89) |  |
| 3 times or more per week | 1999 (37.78) | 590 (48.44) |  |
| 1−2 times per week | 2235 (42.24) | 390 (32.02) |  |
| Hardly ever eat | 542 (10.24) | 81 (6.65) |  |
| Frequency of eating late-night snacks |  |  | <0.001 |
| Every day | 1181 (22.32) | 452 (37.11) |  |
| 3 times or more per week | 863 (16.31) | 267 (21.92) |  |
| 1−2 times per week | 1057 (19.98) | 187 (15.35) |  |
| Hardly ever eat | 2190 (41.39) | 312 (25.62) |  |
| **Family information** |  |  |  |
| Father’s age (years) | 36 (33−39) | 36 (34−39) | <0.001 |
| Mother’s age (years) | 35 (32−37) | 35 (33−38) | <0.001 |
| Paternal BMI (kg/m^2^) | 25.25 (22.86−27.76) | 27.04 (24.62−30.07) | <0.001 |
| Maternal BMI (kg/m^2^) | 22.23 (20.20−24.61) | 23.88 (21.72−27.06) | <0.001 |
| Father's level of education |  |  | <0.001 |
| Secondary school/vocational college and below | 1958 (37.01) | 534 (43.84) |  |
| Bachelor’s degree/diploma | 2895 (54.72) | 639 (52.46) |  |
| Postgraduate and above | 438 (8.28) | 45 (3.69) |  |
| Mother’s level of education |  |  | <0.001 |
| Secondary school/vocational college and below | 1686 (31.87) | 435 (35.71) |  |
| Bachelor’s degree/diploma | 3210 (60.67) | 747 (61.33) |  |
| Postgraduate and above | 395 (7.47) | 36 (2.96) |  |
| Family members with diabetes |  |  | <0.001 |
| 0 | 1125 (21.27) | 434 (35.63) |  |
| 1 | 3810 (72.02) | 642 (52.71) |  |
| 2 | 313 (5.92) | 112 (9.20) |  |
| 3 | 32 (0.60) | 24 (1.97) |  |
| 4 | 7 (0.13) | 4 (0.33) |  |
| 5 | 3 (0.06） | 2 (0.16) |  |
| Household annual income (RMB) |  |  | <0.001 |
| <100,000 | 1889 (35.70) | 466 (38.26) |  |
| 100,000−300,000 | 2382 (45.02) | 591 (48.52) |  |
| 300,000−600,000 | 774 (14.63) | 123 (10.10) |  |
| 600,000−1,000,000 | 175 (3.31) | 19 (1.56) |  |
| ≥1,000,000 | 71 (1.34) | 19 (1.56) |  |

**Abbreviations:** BMI, body mass index.

Continuous variables were tested for normality. Normally distributed variables are presented as mean (standard deviation), while skewed variables are presented as median (interquartile range). Categorical variables are represented as count (percentage, %).

^*^*P value* was calculated using the Student’s t test for normally distributed variables, the rank-sum test for skewed variables, and the Fisher’s exact or χ^2^ test for categorical variables.

**Figure S1.** Comparison of Childhood Obesity Distributions before (Panel A) and after (Panel B) SMOTE Balancing.

**A**

**B**

**Abbreviations:** SMOTE, Synthetic Minority Over-sampling Technique.

**Figure S2.** Distribution Plots for All Features before Multiple Imputation for Missing Data.

**Figure S3.** Relationship Diagrams for All Study Features by Childhood Obesity.

**Figure S4.** Comparison of Distributions before and after Multiple Imputation for Continuous Features.

**Figure S5.** Comparison of Distribution before and after Multiple Imputation for Categorical Features.

**Figure S6.** Spearman Correlation Heatmap.

**Abbreviations:** BMI, body mass index; dmkin, family members with diabetes.

**Table S2.** Performance Evaluation of 12 Machine Learning Models.

| **Model** | **Sensitivity** | **Specificity** | **Accuracy** | **PPV** | **NPV** | **F1-score** | **Youden’s index** | **AUC** |
| --- | --- | --- | --- | --- | --- | --- | --- | --- |
| Gradient Boosting | 0.8086 | 0.9457 | 0.8869 | 0.9180 | 0.8681 | 0.8598 | 0.7543 | 0.9557 |
| Random Forest | 0.7793 | 0.9282 | 0.8643 | 0.8906 | 0.8485 | 0.8312 | 0.7074 | 0.9503 |
| LightGBM | 0.8086 | 0.9413 | 0.8844 | 0.9119 | 0.8676 | 0.8571 | 0.7499 | 0.9491 |
| XGBoost | 0.8125 | 0.9487 | 0.8903 | 0.9224 | 0.8708 | 0.8640 | 0.7612 | 0.9449 |
| Extra Trees | 0.7539 | 0.9018 | 0.8384 | 0.8521 | 0.8300 | 0.8000 | 0.6557 | 0.9252 |
| KNN | 0.5781 | 0.9457 | 0.7881 | 0.8889 | 0.7491 | 0.7006 | 0.5239 | 0.9183 |
| AdaBoost | 0.7617 | 0.8886 | 0.8342 | 0.8369 | 0.8324 | 0.7975 | 0.6503 | 0.9072 |
| QDA | 0.5547 | 0.8827 | 0.7420 | 0.7802 | 0.7253 | 0.6484 | 0.4374 | 0.8317 |
| Decision Tree | 0.6641 | 0.8372 | 0.7630 | 0.7539 | 0.7685 | 0.7061 | 0.5013 | 0.8235 |
| Logistic Regression | 0.5801 | 0.8460 | 0.7320 | 0.7388 | 0.7285 | 0.6499 | 0.4261 | 0.8095 |
| LDA | 0.5840 | 0.8416 | 0.7312 | 0.7346 | 0.7294 | 0.6507 | 0.4256 | 0.8090 |
| Naive Bayes | 0.5527 | 0.8446 | 0.7194 | 0.7275 | 0.7155 | 0.6282 | 0.3973 | 0.7960 |
| Sequential Neural Network Model | 0.6470 | 0.8023 | 0.7357 | 0.7106 | 0.7519 | 0.6772 | 0.4493 | 0.8177 |

**Abbreviations:** AUC, area under the curve; KNN, K-nearest neighbor; LDA, linear discriminant analysis; LightGBM, light gradient boosting machine; PPV, positive predictive value; NPV, negative predictive value; QDA, quadratic discriminant analysis; XGBoost, extreme gradient boosting.

**Figure S7.** Calibration Curves (Panel A) and Clinical Impact Curves (Panel B) for 12 Machine Learning Models.

**A**

**B**

**Abbreviations:** KNN, K-nearest neighbor; LDA, linear discriminant analysis; LightGBM, light gradient boosting machine; QDA, quadratic discriminant analysis; XGBoost, extreme gradient boosting.

**Figure S8.** Confusion Matrices for 12 Machine Learning Models (Panel A) and Sequential Neural Network Model (Panel B).

**A**

**B**

**Abbreviations:** KNN, K-nearest neighbor; LDA, linear discriminant analysis; LightGBM, light gradient boosting machine; QDA, quadratic discriminant analysis; XGBoost, extreme gradient boosting.

**Figure S9.** SHAP Value Heatmap under the Optimal LightGBM Model.

**Abbreviations:** BMI, body mass index; dmkin, family members with diabetes; LightGBM, light gradient boosting machine; SHAP, SHapley Additive exPlanations.
